# Imbalance Between Interleukin-1β and Interleukin-1 Receptor Antagonist in Epicardial Adipose Tissue Is Associated With Non ST-Segment Elevation Acute Coronary Syndrome

**DOI:** 10.3389/fphys.2020.00042

**Published:** 2020-02-05

**Authors:** Valentina Parisi, Laura Petraglia, Serena Cabaro, Vittoria D’Esposito, Dario Bruzzese, Giusy Ferraro, Andrea Urbani, Fabrizio Vincenzo Grieco, Maddalena Conte, Aurelio Caruso, Maria Gabriella Grimaldi, Antonio de Bellis, Salvatore Severino, Pasquale Campana, Emanuele Pilato, Giuseppe Comentale, Maddalena Raia, Giulia Scalia, Giuseppe Castaldo, Pietro Formisano, Dario Leosco

**Affiliations:** ^1^Department of Translational Medical Science, University of Naples Federico II, Naples, Italy; ^2^Institute of Experimental Endocrinology and Oncology, National Research Council, Naples, Italy; ^3^Department of Public Health, University of Naples Federico II, Naples, Italy; ^4^Casa di Cura San Michele, Maddaloni, Italy; ^5^DAI Emergenze Cardiovascolari, Medicina Clinica e dell’Invecchiamento, Università degli Studi di Napoli Federico II, Naples, Italy; ^6^Ceinge Biotecnologie Avanzate s.c. a r.l., Naples, Italy; ^7^Dipartimento di Medicina Molecolare e Biotecnologie Mediche, Università degli Studi di Napoli Federico II, Naples, Italy

**Keywords:** epicardial adipose tissue, inflammation, IL-1, IL1-ra, acute coronary syndrome

## Abstract

**Introduction:**

Interleukin-1beta (IL-1β) is crucially involved in the pathogenesis of coronary atherosclerotic diseases (CAD) and its inhibition has proven cardiovascular benefits. Epicardial adipose tissue (EAT) is a local source of inflammatory mediators which may negatively affect the surrounding coronary arteries. In the present study, we explored the relationship between serum and EAT levels of IL-1β and IL-1 receptor antagonist (IL-1ra) in patients with chronic coronary syndrome (CCS) and recent acute coronary syndrome (ACS).

**Methods:**

We obtained EAT biopsies in 54 CCS (Group 1) and 33 ACS (Group 2) patients undergoing coronary artery bypass grafting. Serum and EAT levels of IL-1β and IL-1ra were measured in all patients. An immunophenotypic study was carried out on EAT biopsies and the CD86 events were studied as markers of M1 macrophages.

**Results:**

Circulating levels of IL-1β were significantly higher in the overall CAD population compared to a control group [7.64 pg/ml (6.86; 8.57) vs. 1.89 pg/ml (1.81; 2.29); *p* < 0.001]. In contrast, no differences were observed for serum IL-1ra levels between CAD and controls. Comparable levels of serum IL-1β were found between Groups 1 and 2 [7.6 pg/ml (6.9; 8.7) vs. 7.9 pg/ml (7.2; 8.6); *p* = 0.618]. In contrast, significantly lower levels of serum IL-1ra were found in Group 2 compared to Group 1 [274 pg/ml (220; 577) vs. 603 pg/ml (334; 1022); *p* = 0.035]. No differences of EAT levels of IL-1β were found between Group 2 and Group 1 [3.4 pg/ml (2.3; 8.4) vs. 2.4 pg/ml (1.9; 8.0); *p* = 0.176]. In contrast, significantly lower EAT levels of IL-1ra were found in Group 2 compared to Group 1 [101 pg/ml (40; 577) vs. 1344 pg/ml (155; 5327); *p* = 0.002]. No correlation was found between EAT levels of IL-1β and CD86 and CD64 events.

**Conclusion:**

The present study explores the levels of IL-1β and IL-1ra in the serum and in EAT of CCS and ACS patients. ACS seems to be associated to a loss of the counter-regulatory activity of IL-1ra against the pro-inflammatory effects related to IL-1β activation.

## Introduction

Inflammation is involved in the pathophysiology of several cardiovascular diseases and clinical and experimental data support its crucial involvement in the pathogenesis and progression of atherosclerotic processes (Ross, 1999; Hansson, 2005; Libby et al., 2009). Interleukin-1beta (IL-1β) is a pro-inflammatory cytokine and its role in promoting inflammation in coronary atherosclerotic diseases (CAD) has been widely explored (Dinarello, 2011). It plays an important role in the inflammatory cascade and coordinates the cellular response to tissue injury promoting the recruitment of inflammatory cells and the increased production of other cytokines (Dinarello, 2011). IL-1β both initiates and propagates several processes leading to the formation, growth, and rupture of the atherosclerotic plaque (Dinarello, 2011; Buckley and Abbate, 2018). The recent results of the Canakinumab Antiinflammatory Thrombosis Outcome Study (CANTOS) strengthen the potential implication of IL-1β in conditioning the clinical course of patients after an acute coronary syndrome (ACS) (Ridker et al., 2017). In fact, this study has demonstrated that targeting the IL-1β innate immunity pathway with canakinumab significantly reduces the recurrence of new cardiovascular events (Ridker et al., 2017).

The IL-1 receptor antagonist (IL-1ra) is the endogenous counter-regulator of IL-1 and can serve as a detectable surrogate parameter for high IL-1β activity (Arend et al., 1998).

In patients with stable CAD, epicardial adipose tissue (EAT), the visceral fat depot of the heart, secretes IL-1β and the levels of this cytokine in cardiac visceral fat have been found to be higher than in subcutaneous adipose tissue and independent from circulating inflammatory markers (Mazurek et al., 2003). Furthermore, EAT is a local source of other inflammatory mediators that are involved in the pathogenesis of coronary atherosclerosis. Given the tight anatomical connection with epicardial coronary arteries, EAT could promote atherosclerosis through vasocrine and paracrine mechanisms (Mazurek et al., 2003; Hirata et al., 2011a). Dynamic changes of EAT inflammatory profile seem to contribute to atherosclerotic plaque instability and different evidence support a close association between a pro-inflammatory EAT phenotype and ACS (Mazurek et al., 2003; Pedicino et al., 2017). The aim of the present study is to investigate the behavior of IL-1β and IL-1ra, at serum and EAT levels, in patients referred to elective coronary artery by-pass grafting (CABG) for a chronic coronary syndrome (CCS) resistant to optimized medical therapy, and in patients referred to urgent CABG after a recent non-ST-segment elevation ACS syndrome.

## Materials and Methods

### Study Population

The patient population included 87 CAD patients undergoing CABG. The study group was composed of: (a) Group 1: CCS patients with hemodynamically significant coronary stenosis in the presence of limiting angina or angina equivalent, with insufficient response to optimized medical therapy, referred to elective CABG (n. 54 pts) (Knuuti et al., 2019); (b) Group 2: ACS patients referred to urgent CABG within 1 week from non-ST-segment elevation myocardial infarction (n. 33 pts) (Roffi et al., 2016). Exclusion criteria were: (a) CAD patients with other pathologic conditions associated with morphologic and functional EAT changes, such as aortic valve stenosis (Parisi et al., 2015; Davin et al., 2019) and atrial fibrillation (Christopher et al., 2017); (b) hemodynamic instability; (c) CAD patients with cancer and or systemic inflammatory diseases which might affect EAT and/or circulating inflammatory profile; (d) CAD patients with a recent myopericarditis.

Before cardiac surgery, all patients underwent a complete clinical examination with assessment of cardiovascular risk factors and drug therapies, and a standard echocardiographic examination including EAT measurement. Echocardiograms were performed by a VIVID E9 (GE Healthcare) machine, according to standard techniques. EAT thickness was obtained from a parasternal long-axis view. EAT was visualized in parasternal long-axis view between the free wall of the right ventricle and the anterior surface of the ascending aorta. Once visualized the EAT deposit, the maximum EAT thickness was measured at end-systole, as we have previously described (Parisi et al., 2019b). The average value from three cardiac cycles was used for the statistical analysis.

The study was approved by the local Ethics Committee. All procedures performed in the study were in accordance with the ethical standards of the institutional or national research committee and with the 1964 Helsinki declaration and its later amendments or comparable ethical standards and conformed to the Declaration of Helsinki on human research. All patients included in the study gave written informed consent after receiving an accurate explanation of the study protocol and of the potential risks related to the procedures adopted by the study.

### Tissues Collection

Before cardiac surgery, we collected blood samples for serum IL-1β and IL-1ra determination. Intraoperative EAT biopsies (average 0.1–0.5 g) before the initiation of cardiopulmonary bypass. EAT biopsies were taken between the free wall of the right ventricle and the anterior surface of the ascending aorta, just after the opening of the pericardial sac.

### Cytokine and Growth Factor Assay

Epicardial adipose tissue secretomes were obtained as follows: tissues were weighted, cut into small pieces, and transferred into a 12-well plate. According to tissue weight, serum-free Dulbecco modified Eagle medium (DMEM)-F12 (1:1) containing 0.25% BSA (1 ml medium/0.1 g tissue) was added to the well and incubated at 37°C in a CO_2_ incubator. After 24 h, medium was collected and centrifuged at 14,000 × *g* to remove debris and analyzed for cytokines content. EAT conditioned media and serum were screened for the concentration of IL-1β and IL-1ra using the Bioplex Multiplex human cytokine assay (Bio-Rad, Hercules, CA, United States), as previously reported (Parisi et al., 2015; Cabaro et al., 2018).

We also measured serum levels of IL-1β and of IL-1ra in a control group of 77 subjects without history and/or signs and symptoms suggestive of CAD, cancer, and systemic inflammatory diseases.

### Cytofluorometry

The freshly picked EAT bioptic fragments was stored in PBS (Phosphate Buffered Saline), an isotonic saline solution non-toxic to cells, and sent to the Flow Cytometry laboratory for preparation and analysis. Samples were then processed in order to obtain an homogeneous cell suspension. For this purpose, mechanical exfoliation of the bioptic fragment was performed through the use of a scalpel (or slides), with addition of PBS with FBS (1%) to facilitate its disintegration. Therefore, the resuspended cells in solution were centrifuged at 1600 rpm for 10 min. Afterward, the pellet was recovered and a quantity of 1% FBS PBS was added to obtain a right concentration of cells. The sample obtained was aliquoted in cytometry tubes and incubated with adequate monoclonal antibodies (MoAb) concentrations for 20 min at 4°C in the dark. After incubation, the possibly present red cells were removed using a hypotonic solution (ammonium chloride).

The sample was left for 15–20 min at room temperature and in the dark. The cells were subsequently centrifuged at 2000 rpm for 3 min (Eppendorf 5804 centrifuge) and the resulting pellet was resuspended in 200 μL of PBS or FACS Flow. Finally, the cells were acquired by the BD FACS-Canto II flow cytometer and the data were subsequently analyzed with the aid of the FACS-Diva software (BD). The immunophenotypic study was carried out by setting up multiparametric panels, which allowed the evaluation of macrophages. The antigens CD86 and CD64 were studied as markers of M1 macrophages.

### Statistical Analysis

All statistical analyses have been performed using R platform (version 3.5.0). Data were expressed as absolute frequencies and percentages in case of categorical factors and as mean ± standard deviation with range or median [25th; 75th percentile] in case of numerical variables. The latter description was preferred in case of variables showing a consistent asymmetry. Accordingly, comparisons between groups were based either on the Chi-square test, the fisher exact test (when appropriate), the *t*-test for independent samples, and the Mann–Whitney *U*-test. Correlations among variables were assessed using the non-parametric Spearman rank correlation. A two-tailed *p*-value of less than 0.05 was considered statistically significant in all analyses.

## Results

Table 1 illustrates demographic, clinical, and echocardiographic characteristics of the study population. In the overall CAD population, the mean age was 65.4 ± 9.2 (range 45–84) years and 24.1% of patients were females. Fifty percent of patients were diabetics, 82.8% had hypertension, 47.7% were smokers, and 64% had dyslipidaemia. Mean left ventricular ejection fraction (LVEF) was 56.8 ± 10.7%. Mean EAT thickness was 11 ± 3.3 mm.

**TABLE 1:** Demographic and clinical data of the study population.

	**Overall (*n* = 87)**	**Group 1 (*n* = 54; 62.1%)**	**Group 2 (*n* = 33; 37.9%)**	***p*-value**
Age (years)	65.4 ± 9.2 (45–84)	65.9 ± 8.6 (50–83)	65.7 ± 10.2 (45–84)	0.942
Gender, female	21 (24.1)	13 (24.1)	8 (24.2)	1.000
BMI	28.2 ± 4.4 (19.1–41.2)	28 ± 4.2 (19.7–39.1)	28.3 ± 4.6 (19.1–41.2)	0.826
Hypertension	72 (82.8)	46 (85.2)	26 (78.8)	0.560
Diabetes	44 (50.6)	25 (46.3)	19 (57.6)	0.379
Dyslipidemia	55 (64)	38 (71.7)	17 (51.5)	0.068
Smokers, *n* (%)	41 (47.7)	23 (43.4)	18 (54.5)	0.377
BBs, *n* (%)	78 (91.8)	50 (92.6)	28 (90.3)	0.702
Aspirin, *n* (%)	82 (96.5)	52 (96.3)	30 (96.8)	1
Ace-i/Arbs, *n* (%)	63 (74.1)	38 (70.4)	25 (80.6)	0.441
Statins, *n* (%)	71 (83.5)	42 (77.8)	29 (93.5)	0.073
Troponin rise, *n* (%)	28 (36.4)	0 (0)	28 (90.3)	<0.001
E/A	0.9 [0.8; 1.1] (0.6–4.1)	0.9 [0.8; 1.4] (0.6–4.1)	0.8 [0.8; 1] (0.7–1.1)	0.378
E/e’	10 [7.4; 13.6] (4.8–21)	9.7 [7.3; 13.9] (4.8–21)	10 [7; 11.7] (6–24)	0.847
LVTSd (mm)	33.5 [30.5; 39.8] (26–54)	32 [28; 35] (26–47)	45 [34; 51.5] (33–54)	0.031
LVTDd (mm)	49 [45; 53] (37–62)	48 [43; 51] (40–56)	55 [46.5; 57] (37–62)	0.041
Septum (mm)	9.5 [8; 11] (6–14)	9 [7.8; 10.2] (6–12)	10 [8; 12] (7–14)	0.148
Pp (mm)	9 [7; 10] (6–13)	9 [7; 10.2] (6–13)	9.5 [7.8; 10] (6–11)	0.644
LV mass-i (g/m^2^)	74.6 [61.4; 96.2] (0.7–136)	69.7 [61.4; 84.7] (1–131.1)	81.5 [32.1; 108.1] (0.7–136)	0.582
RWT	0.35 [0.3; 0.45] (0.24–0.54)	0.38 [0.28; 0.46] (0.25–0.49)	0.33 [0.27; 0.45] (0.24–0.54)	0.811
LVEF (%)	56.8 ± 10.7 (30–81)	59.7 ± 10.3 (35–81)	52.4 ± 10.8 (30–65)	0.003
EAT thickness (mm)	11 ± 3.3 (0–18)	10.8 ± 4.2 (0–18)	12.5 ± 2 (9–15)	0.169

*Data are expressed as *n* (%) or as mean ± standard deviation (min–max) or median [25th–75th percentile] (min–max). BMI, body mass index; BBs, beta-blockers; ACE-I, angiotensin converting enzyme inhibitors; ARB, angiotensin receptor blockers; LVTDd, left ventricular tele-diastolic diameter; LVTSd, left ventricular tele-sistolic diameter; Pp, posterior wall; LV mass-I, left ventricular mass-index; RWT, relative wall thickness; LVEF = left ventricular ejection fraction; EAT, epicardial adipose tissue.*

**FIGURE 1:**
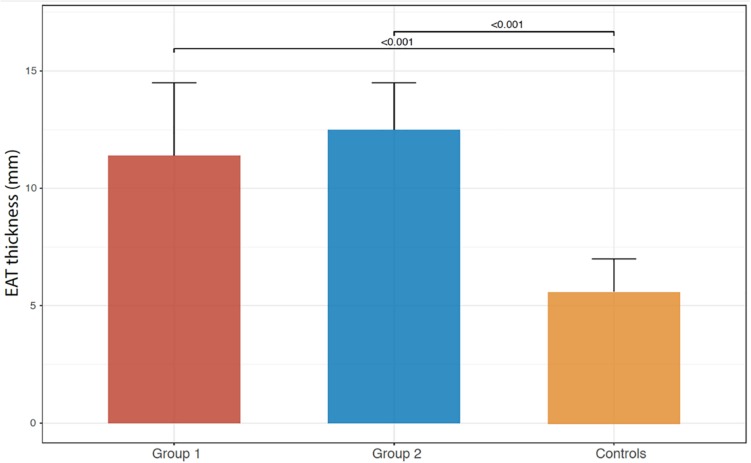
Bar graphs illustrating EAT thickness in Groups 1 and 2 and controls.

Between Groups 1 and 2, no differences were found in demographic and clinical data. Group 2 showed signs of the recent ACS with increased left ventricular end-diastolic and end-systolic diameters (*p* = 0.031 and *p* = 0.041, respectively) compared to Group 1 and decreased LVEF (*p* = 0.003), as expected. EAT thickness values of both Groups 1 and 2 were significantly higher than those measured in a control group of subjects without history and/or signs and symptoms suggestive of CAD, cancer, and systemic inflammatory diseases (10.8 ± 4.2 mm and 12.5 ± 2 mm vs. 5.6 ± 1.4; *p* < 0.001 for both comparisons) (Figure 1). There was a slight, but not significant, increase of EAT thickness in Group 2 compared to Group 1.

### Serum Levels of IL-1β and IL-1ra

**FIGURE 2:**
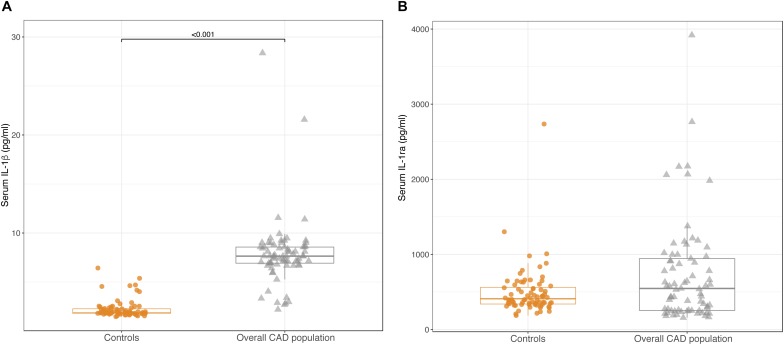
Box-plots showing serum levels of IL-1β **(A)** and IL-1ra **(B)** in control subjects and in the overall CAD population. Boxes are defined by Q1, median (bold line) and Q3. Whiskers reach the minimum and the maximum of the distribution except for the presence of outliers, defined as data points below Q1 – 1.5 × IQR or above Q3 + 1.5 × IQR. To avoid overlapping a small amount of horizontal jitter was added. Q1, first quartile; Q3, third quartile; IQR = Q3 – Q1.

In the whole population of CAD patients, circulating levels of IL-1β were significantly higher than in controls [7.64 pg/ml (6.86; 8.57) vs. 1.89 pg/ml (1.81;2.29); *p* < 0.001] (Figure 2A). In contrast, no differences were observed for IL-1ra levels between CAD patients and controls [547 pg/ml (253; 957) vs. 410 pg/ml (335; 573); *p* = 0.199] (Figure 2B).

**FIGURE 3:**
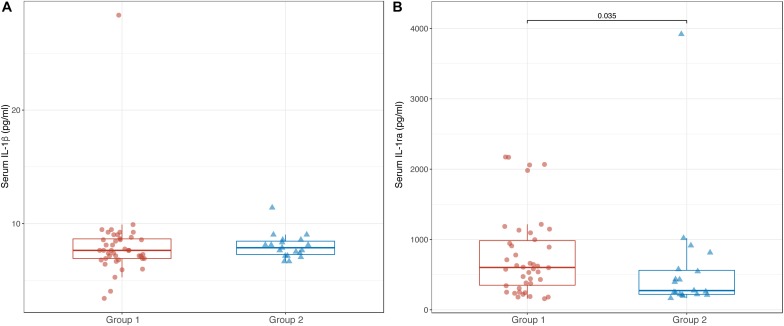
Box-plots showing serum levels of IL-1β **(A)** and IL-1ra **(B)** in Groups 1 and 2. Boxes are defined by Q1, median (bold line) and Q3. Whiskers reach the minimum and the maximum of the distribution except for the presence of outliers, defined as data points below Q1 – 1.5 × IQR or above Q3 + 1.5 × IQR. To avoid overlapping a small amount of horizontal jitter was added. Q1, first quartile; Q3, third quartile; IQR = Q3 – Q1.

Comparable levels of serum IL-1β were found between Groups 1 and 2 [7.6 pg/ml (6.9; 8.7) vs. 7.9 pg/ml (7.2; 8.6); *p* = 0.618] (Figure 3A). In contrast, serum levels of IL-1ra were significantly lower in Group 2 than in Group 1 [274 pg/ml (220; 577) vs. 603 pg/ml (334; 1022); *p* = 0.035] (Figure 3B).

In the overall population of CAD patients, there was no correlation between serum levels of IL-1β and IL-1ra (*r* = 0.135; *p* = 0.254). Accordingly, no significant correlation was found between serum levels of IL-1β and IL-1ra in both Groups 1 and 2 (*r* = 0.172; *p* = 0.276 and *r* = −0.239; *p* = 0.324, respectively).

Overall, these results show an exalted circulating inflammatory profile of CAD patients compared to a control population, as indicated by the increased serum IL-1β concentrations. Interestingly, the different behavior of serum IL-1ra in Groups 1 and 2, begins to delineate differences between stable CCS patients and ACS patients characterized by the loss of the counter-regulatory activity of IL-1ra against the potent inflammatory stimulation induced by IL-1β.

### EAT Levels of IL-1β and IL-1ra

**FIGURE 4:**
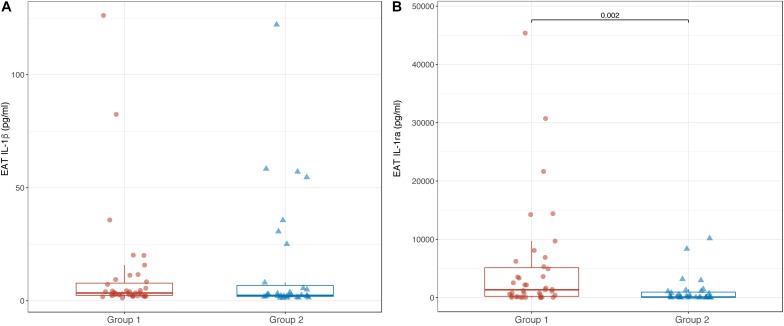
Box-plots showing EAT levels of IL-1β **(A)** and IL-1ra **(B)** in Groups 1 and 2. Boxes are defined by Q1, median (bold line) and Q3. Whiskers reach the minimum and the maximum of the distribution except for the presence of outliers, defined as data points below Q1 – 1.5 × IQR or above Q3 + 1.5 × IQR. To avoid overlapping a small amount of horizontal jitter was added. Q1, first quartile; Q3, third quartile; IQR = Q3 – Q1.

Epicardial adipose tissue levels of IL-1β were not different between Groups 1 and 2 [3.4 pg/ml (2.3; 8.4) vs. 2.4 pg/ml (1.9; 8.0); *p* = 0.176]. In contrast, EAT levels of IL-1ra were significantly lower in Group 2 compared to Group 1 [101 pg/ml (40; 577) vs. 1344 (155; 5327); *p* = 0.002] (Figures 4A,B). As well as for the serum, in the overall CAD population, there was no correlation between EAT levels of IL-1β and IL-1ra (*r* = 0.165; *p* = 0.154). Accordingly, no significant correlation was found between serum levels of IL-1β and IL-1ra in both Groups 1 and 2 (*r* = 0.026; *p* = 0.875 and *r* = 0.313; *p* = 0.087, respectively).

Of interest, in the overall population of CAD patients, there was a significant correlation between serum and EAT levels of IL-1ra (*r* = 0.679; *p* < 0.001). This correlation was also confirmed in the two separate CAD groups (Group 1: *r* = 0.611; *p* < 0.001; Group 2: 0.675; *p* = 0.002).

Overall these results confirm, also at EAT level, the loss of the counteregulatory activity of IL-1ra.

### M1 Macrophages and EAT IL-1β Levels

Epicardial adipose tissue inflammation in CAD patients has been largely ascribed to macrophage polarization. Previous studies have reported a direct correlation between macrophage M1 polarization and the EAT levels of inflammatory cytokines (Hirata et al., 2011b). Therefore, we tested in the present population the correlation between IL-1β levels and CD86 and CD64 as markers of M1 macrophages. However, there were no significant correlations between IL-1β levels [2.9 pg/ml (1.2–126.2)] and CD86 events [n. 3 (0–17); *r* = −0.051; *p* = 0.935) and CD64 events [n. 15 (3–98); *r* = −0.31; *p* = 0.564).

## Discussion

The main findings of the present study were: (1) CAD patients showed higher EAT thickness and circulating levels of IL-1β compared to controls; (2) despite similar circulating levels of IL-1β, ACS patients showed lower serum and EAT levels of IL-1ra compared to CCS patients; (3) serum and EAT levels of IL1-ra were directly correlated; (4) there was no correlation between IL-1β and IL-1ra levels either at local or at systemic levels.

Accumulating evidence support the crucial involvement of inflammation in the pathogenesis of coronary syndromes (Kofler et al., 2005). In this regard, several studies have highlighted the key role of IL-1β in the atherosclerotic process, given the ability of this cytokine to induce a network of pro-inflammatory signaling pathways (Tedgui and Mallat, 2006; Kim et al., 2010). In the present study, we have reported that an imbalance between IL-1β and IL-1ra levels can discriminate patients with CCS and ACS. To note, it has been reported that 5–100-fold greater amounts of IL-1ra are necessary to obtain a 50% inhibition of IL-1β stimulation (Arend et al., 1990). Therefore, even a little reduction il IL-1ra could be associated to a substantial increase of IL-1β activity. We have also shown that CAD patients have higher serum levels of IL-1β, but similar levels of IL-1ra, compared to controls. This evidence can support the hypothesis that higher levels of IL-1β, not paralleled by the increase of IL-1ra at systemic and local levels, may trigger pro-inflammatory and pro-atherosclerotic processes able to promote CAD progression, atherosclerotic coronary plaque instability, and negative left ventricular remodeling after ACS. The increase of EAT thickness observed in CAD patients compared to controls, also confirm previous evidence indicating a direct association between EAT accumulation and its exalted inflammatory status in the CAD population (Picard et al., 2014).

It is widely recognized that IL-1β is responsible of some of the typical features of the unstable atehrosclerotic plaque, such as macrophage/monocyte infiltration and decrease in α-smooth muscle actin (Isoda et al., 2004). In addition, this cytokine may be involved in many of the pathophysiological events leading to ACS occurrence, through the induction of vascular smooth muscle proliferation and neointima formation, impaired vasodilation, increased oxidative stress and procoagulant activity (Bevilacqua et al., 1984; Libby et al., 1988; Chamberlain et al., 2006; Chamberlain et al., 2009). On the other hand, enhanced IL-1β levels are involved in left ventricular remodeling after myocardial infarction. In particular, this cytokine plays an orchestrating role in the inflammatory response to myocardial injury, including enhanced synthesis of other proinflammatory mediators, activation of profibrotic pathways (Hwang et al., 2001), promotion of cytokine-induced cardiomyocyte apoptosis (Bujak and Frangogiannis, 2009), and also exerting a direct negative inotropic effect on myocyte contractility (Gulick et al., 1989).

We also found a significant correlation between circulating and EAT levels of IL-1ra in both CCS and ACS patients. This observation could pave the way for future studies exploring the potential use of circulating IL-1ra as a potential biomarker able to reflect the EAT inflammatory status. Interestingly, in ACS patients, IL-1ra levels were almost twofold lower in the serum and 10-fold lower in EAT compared to CCS patients. This finding indicates that EAT not only reflects the systemic pro-inflammatory status but it also plays an active role in modulating the local inflammatory activity.

Several evidence support the role of EAT in the pathogenesis of cardiovascular diseases (Mazurek et al., 2003; Hirata et al., 2011a; Parisi et al., 2015; Moreno-Santos et al., 2019) and propose EAT as a new therapeutic target (Parisi et al., 2019a). Pedicino et al. (2017) demonstrated the presence of bacterial DNA directly into EAT, surrounding diseased coronary arteries, of patients with ACS. This microbial colonization was associated with up-regulation of NOD-like receptor protein inflammasome involving IL-1β pathway activation in EAT.

Our study report increased circulating levels of IL-1β in both CCS and ACS patients compared to controls, thus confirming the potential implication of this cytokine pathway activation in CAD syndromes. However, the novel finding of the present investigation is the imbalance between IL-1β and IL-1ra levels in patients with recent ACS. In fact, in this population, we found a reduction of IL-1ra, both at systemic and EAT levels, suggesting a loss of the counter-regulatory activity of this cytokine against the pro-inflammatory effects of IL-1β. In this regard, it has been reported a different receptorial affinity of IL-1β and IL-1ra (Arend et al., 1990), thus low reduction of IL-1ra levels is needed to enhance the IL-1β activity. This also explains our findings regarding the lack of correlation between the concentrations of these two cytokines, both in EAT and in the serum.

Our results are in contrast with those reported by Patti et al. (2004, 2005), who showed a serum increase of IL-1ra in patients with ACS, that the authors interpreted as a response to increased IL-1 activity. However, the intercorrelation between biological activities of IL-1β and IL-1ra in the context of ACS are still poorly explored and further studies are needed to definitively assess the dynamic changes of IL-1β and IL-1ra activities in the acute setting.

As regard the relationship between EAT macrophage infiltration and pro-inflammatory cytokines secretion, Hirata et al. (2011b) reported in stable CAD patients that the EAT infiltration and macrophage M1 polarization were directly associated to inflammatory cytokines levels. In the present study, we did not find a correlation between M1 macrophage markers (CD86 and CD64) and EAT levels of IL-1β. A plausible explanation for this finding could be that the number of inflammatory cells in EAT might not necessarily reflect their increased activity. Therefore, an increase of IL-1β levels could not necessarily correspond to an increase of macrophage recruitment and polarization (He et al., 2019). Furthermore, adipocytes secrete cytokines (Yin et al., 2014), thus these cells could be a further source of inflammatory molecules and contribute to the overall changes of cytokine levels found in EAT.

### Study Limitations

Although the results of this study are novel and report for the first time the behavior of IL-1β and its receptor antagonist at EAT and serum levels in CCS and ACS patients, it arises unanswered questions. A first issue regards the quantification of IL-1β levels. Previous evidence has reported the technical difficulties for IL-1β detection, related to the significant amount of pro-IL-l β remaining inside the cells and the binding of this cytokine to large proteins (Borth et al., 1990). Therefore, the expected further increase of IL-1β levels in ACS patients might have been masked.

Another unanswered issue is whether changes of EAT secretory profile in CAD patients might only reflect a status of generalized inflammation or whether the cardiac visceral fat might play an active role in modulating the inflammatory cascade at myocardial level. In this regard, in our CAD population, EAT absolute concentrations of IL-1β and IL-1ra were different from those measured in the serum. In particular, in ACS patients, the IL-1ra reduction in EAT was fivefold higher than that observed in the serum. These findings clearly support the independent role of EAT in the modulation of the inflammatory events associated to ACS.

The last issue regard the timing of serum cytokines determination. In this study, we looked at cytokine levels after myocardial infarction, thus, we cannot exclude that changes of IL-1β and IL-1ra could be the results of a generalized inflammatory response to myocardial injury, thus being the consequence and not the cause of ACS. However, the primary objective of this paper was to strengthen the concept that EAT might reflect the status of systemic inflammation associated to ACS and potentially contribute, given its tight connection with the heart, to myocardial damage through the local release of pro-inflammatory molecules. This activity could represent a trigger for atherosclerotic plaque instability in the first phase of ACS syndrome and, then, contribute to negative LV remodeling after myocardial infarction.

## Conclusion

The present study has explored, for the first time, the balance between IL-1β and IL-1ra in the serum and in EAT of CAD patients with and without a recent ACS. The main finding of the study is that, ACS seems to be associated to the loss of the counter-regulatory activity of IL-1ra against the potent inflammatory effects related to IL-1β activation. Further studies are needed to assess the dynamic changes of these two cytokines during ACS and to define a potential therapeutic role of IL-1β inhibition in the acute setting.

## Data Availability Statement

The datasets generated for this study are available on request to the corresponding author.

## Ethics Statement

The studies involving human participants were reviewed and approved by the Ethics Committee of the University of Naples Federico II. The patients/participants provided their written informed consent to participate in this study.

## Author Contributions

VP, LP, and DL contributed to the conception and design of the study. VD’E, SC, GF, AB, and SS organized the database. DB performed the statistical analysis. FG, MC, AC, MR, GS, and MG wrote the first draft of the manuscript. VP, PC, EP, GCo, GCa, PF, and DL wrote the sections of the manuscript. All authors contributed to the manuscript revision, read, and approved the submitted version.

## Conflict of Interest

The authors declare that the research was conducted in the absence of any commercial or financial relationships that could be construed as a potential conflict of interest.
